# Response to: A Commentary on “Antipsychotic-Induced Parkinsonism is Associated with Working Memory Deficits in Schizophrenia-Spectrum Disorders”

**DOI:** 10.3389/fnbeh.2015.00210

**Published:** 2015-08-12

**Authors:** Stéphane Potvin, Andràs Tikàsz

**Affiliations:** ^1^Centre de Recherche de l’Institut Universitaire en Santé Mentale de Montréal, Montreal, QC, Canada; ^2^Department of Psychiatry, Faculty of Medicine, University of Montreal, Montreal, QC, Canada

**Keywords:** schizophrenia, parkinsonism, extrapyramidal symptom, dosage equivalency, cognition

We read with interest the commentary from Drs Salem and Moustafa (2015) on our paper and we would like to take the opportunity to further discuss two issues, namely, the use of chlorpromazine equivalents (CPZs) to control for the effects of antipsychotics and the need for longitudinal studies examining the complex relationships between cognition and antipsychotic-induced extrapyramidal symptoms (EPS) in schizophrenia.

Drs Salem and Moustafa suggested calculating CPZs as means to rule out confounding dosage and variations in drugs. Although we agree with the notion put forward by the authors, especially in the context of a study investigating the side effects of antipsychotics, we would like to reiterate that there is yet to be a widely accepted robust antipsychotic dosage comparison system that reliably yields consistent equivalences across drugs (Patel et al., 2013). CPZs can be obtained via one of three calculations described by Patel et al. (2013): the mean effective dose in flexible-dose studies, the minimum effective dose, and the consensus of experts method. These methods yield inconsistent results particularly in the case of antipsychotics with low affinities for dopamine-D_2_ receptors (Sweileh et al., 2014). In our study, out of 82 schizophrenia-spectrum patients, 24 patients were treated with clozapine and 18 with quetiapine. In the literature, we note significant fluctuations in CPZs for quetiapine (60–175.5 mg/day) and clozapine (50–138.8 mg/day), an ambiguity highlighted when the two drugs are considered relative to one another (see Table S1 in Supplementary Material). In fact, depending on the CPZ calculation method, quetiapine will be considered more or less potent than clozapine. Therefore, even when only one equivalence system is employed, the comparison of antipsychotic medication dosage between studies for an individual drug is troublesome, and interpreting between-drug differences is problematic. The limited number of studies comparing directly atypical antipsychotics to chlorpromazine, an older typical antipsychotic, might explain some of the variability between the CPZ calculations. Nevertheless, when examining olanzapine equivalents, a system based on the availability of a larger set of randomized-controlled trials (RCTs) (Leucht et al., 2014, 2015), significant discrepancies between calculation methods of over 30% can be observed for both quetiapine (20–32.3 mg/day) and clozapine (30.6–40 mg/day) (see Table S1 in Supplementary Material). Other than CPZ, Daily Defined Doses (DDD) is a popular platform developed to compare drug utilization (Danivas and Venkatasubramanian, 2013). It is not, however, a measure of therapeutic efficacy like CPZ (Patel et al., 2013). Finally, equivalents of dopamine-D_2_ receptor occupancy appears as a relevant system amongst the remaining alternatives, as it targets a key neuropharmacological mechanism involved in the emergence of antipsychotic-induced EPS (de Greef et al., 2011). Indeed, several positron emission tomography studies have shown that antipsychotics are more likely to produce EPS at dosages associated with striatal D_2_ occupancy higher than 80% (Kapur and Seeman, 2001). However, the role of D_2_ receptors in the pathophysiology of EPS (dystonia, dyskinesia, akathisia) other than parkinsonism is less clear (Mehta et al., 2015). Moreover, antipsychotics bind several receptors other than D_2_ contributing to, or preventing, the emergence of EPS, such as dopamine D_3_, D_4_, serotonin 5-HT_2A_, and α_1_-adrenergic receptors (Horacek, 2000). For these reasons, the D_2_ equivalency method can be reductionist. In sum, until a consistent reliable system is developed and implemented, utilizing one dosage equivalence system versus another is likely to introduce significant caveats in the interpretation of experimental results, especially in the case of studies including patients treated with clozapine or quetiapine.

The second issue that we wanted to raise concerns the fact that most studies investigating the relationship between cognition and EPS in schizophrenia are based on cross-sectional designs, which are more vulnerable to confounding factors than longitudinal designs. As rightly mentioned by Drs Salem and Moustafa, we sought to overcome this problem in our study by performing hierarchical multivariate linear regression analyses incorporating several socio-demographic and psychiatric variables likely to influence the cognition–EPS association (Potvin et al., 2015). Still, we feel that longitudinal studies are required in order to clarify the temporal dynamics of the association we observed between cognition (e.g., working memory) and drug-induced Parkinsonism. Interestingly, several RCTs have examined the effects of antipsychotics on cognition, which provide an opportunity to study the cognition–EPS association in a longitudinal fashion. The study of the cognition–EPS association is particularly relevant in this context, since it is theoretically possible that the presumed beneficial effects of second-generation antipsychotics (SGAs) on cognition are actually explained by the lower liability of these drugs to induce EPS, compared to first-generation antipsychotics (Leucht et al., 2013). It must also be considered that by causing fewer EPS, the treatment with SGAs renders the prescription of adjuvant anticholinergic drugs less necessary, the latter being well-known to impair cognition (Bubser et al., 2012). Recent meta-analyses have shown that the beneficial effects of SGAs (risperidone and olanzapine) on cognition are small-to-moderate (Desamericq et al., 2014). These benefits may not only reflect true effects but also be confounded by the effects of EPS and/or anticholinergics. Therefore, we reviewed 35 RCTs included in a recent meta-analysis from Nielsen et al. (2015) on the effects of antipsychotic on cognition in schizophrenia and systematically retrieved information on anticholinergics, EPS scales, EPS–cognition associations, and cognitive tests (see Table 1). Of the 35 RCTs reviewed, 12 studies did not measure EPS. Of the remaining 23 studies, only 14 performed statistics on potential associations between EPS and cognition. Of these, six trials found an association between EPS and cognition, and eight did not. Although such results do not firmly establish a relationship between EPS and cognition, they do not rule it out either, especially since the mean duration of trials was 24 weeks, which may not be long enough to detect associations between EPS and cognition in schizophrenia. To our knowledge, the most reliable associations highlighted in cross-sectional studies are between cognition and tardive dyskinesia (Pantelis et al., 2001; Wu et al., 2013), which typically occurs later during treatment than akathisia, dystonia, and parkinsonism. Also of methodological concern, among the 35 RCTs reviewed, only 2 studies excluded anticholinergic drugs (Note: 8 trials did not report information on anticholinergics). Moreover, of the 25 remaining trials, only 9 studies examined potential associations between changes in cognitive performance and the prescription of adjuvant anticholinergics. At a time when the pharmacological treatment of cognitive deficits in schizophrenia is recognized as a top clinical priority, the lack of knowledge on the above-mentioned factors impedes our ability to reach valid conclusions on the presumed superior efficacy of SGAs (mostly risperidone and olanzapine) on cognition in schizophrenia. In return, these limitations undermine the ongoing debate about the pharmacological mechanisms that should be targeted to improve cognitive performance in schizophrenia.

**Table 1:** **Review of the studies included in the meta-analysis from Nielsen et al. (2015)**.

Reference	Antipsychotics	Duration (weeks)	*N*	AntiCh	AnticCh– cognition association	EPS scale	EPS– cognition association	Cognitive functions
Bellack et al. (2004)	Clozapine, risperidone	29	22	–	–	–	–	–
Bender et al. (2006)	Clozapine, olanzapine	26	31	Allowed	–	SAS	No	Executive functioning
Bilder et al. (2002)	Clozapine, olanzapine, risperidone, haloperidol	14	101	Allowed	Yes	ESRS	Yes	Composite index (*general executive and perceptual organization, declarative verbal learning and memory, processing speed and attention, simple motor functioning*)
Buchanan et al. (1994)	Clozapine, haloperidol	10	38	Allowed	–	SAS	Yes	Visual memory
Davidson et al. (2009)	Amisulpride, quetiapine, ziprasidone, haloperidol	26	320	Allowed	–	SHRS	–	–
Gallhofer et al. (2007)	Sertindole, haloperidol	12	32	Excluded	–	SAS	No	Speed and attention, executive functioning
Gurpegui et al. (2007)	Olanzapine, risperidone	48	163	Allowed	–	–	–	–
Harvey et al. (2008)	Clozapine, ziprasidone	12	100	–	–	–	–	–
Harvey et al. (2003)	Olanzapine, risperidone	8	267	Allowed	No	ESRS	Yes	Verbal memory, vigilance
Harvey et al. (2006a)	Olanzapine, ziprasidone	26	72	–	–	ESRS	No	Attention, task switching, verbal memory, executive functioning, verbal fluency
Harvey et al. (2006b)	Quetiapine, risperidone	8	289	–	–	–	–	–
Honer et al. (2006)	Clozapine + risperidone or clozapine + placebo	8	65	Allowed	–	ESRS	–	–
Jerrell and Ramirez (2008)	Olanzapine, risperidone, typical antipsychotic drugs	52	108	Allowed	No	–	–	–
Keefe et al. (2007b)	Olanzapine, quetiapine, risperidone	52	81	Allowed	No	SAS	No	Composite index (*processing speed, reasoning and problem solving, verbal memory and working memory, vigilance*)
Keefe et al. (2007a)	Olanzapine, quetiapine, risperidone, ziprasidone, perphenazine	78	128	Allowed	Yes	SAS	Yes	Composite index (*processing speed, reasoning, working memory, verbal memory, vigilance*)
Krakowski et al. (2008)	Clozapine, olanzapine, haloperidol	12	100	Allowed	–	ESRS	No	Composite index (*motor, executive function, verbal memory, visual memory, visuospatial ability, visual motor tracking, attention*)
Lee et al. (1999)	Clozapine, Typical antipsychotic drugs	52	52	Allowed	No	SAS	Yes	Verbal fluency, executive functioning, verbal working memory
Lee et al. (2007)	Risperidone, haloperidol	8	20	–	–	–	–	–
Lindenmayer et al. (2007)	Olanzapine, haloperidol	12	33	Allowed	–	SAS	–	–
Ljubin et al. (2000)	Olanzapine, fluphenazine	22	18	–	–	–	–	–
McGurk et al. (2005)	Clozapine, risperidone	29	35	–	–	–	–	–
Meltzer et al. (2008)	Clozapine, olanzapine	26	24	Allowed	–	SAS	–	–
Mori et al. (2004)	Olanzapine, perospirone, quetiapine, risperidone	12	77	Allowed	Yes	–	–	–
Mortimer et al. (2007)	Amisulpride, olanzapine	26	18	Excluded	–	AIMS	–	–
Muscatello et al. (2011)	Aripiprazole + clozapine or placebo + clozapine	24	31	–	–	–	–	–
Ernst Nielsen et al. (2014)	Clozapine + sertindole or clozapine + placebo	12	50	Allowed	–	–	–	–
Purdon et al. (2000)	Olanzapine, risperidone, haloperidol	54	65	Allowed	Yes	ESRS	No	Composite index (*motor skills, attention, verbal fluency, reasoning, non-verbal fluency and construction, executive functioning, immediate recall*)
Purdon et al. (2001)	Quetiapine, haloperidol	26	25	Allowed	–	SAS	–	–
Riedel et al. (2007a)	Olanzapine, quetiapine	8	33	Allowed	–	ESRS	–	–
Riedel et al. (2007b)	Quetiapine, risperidone	12	34	Allowed	–	SAS	–	–
Sergi et al. (2007)	Olanzapine, risperidone, haloperidol	8	73	Allowed	–	–	–	–
Smith et al. (2001)	Olanzapine, haloperidol	21	29	Allowed	No	SAS	No	Verbal and visual recall and memory, executive functioning, verbal fluency
van Veelen et al. (2010)	Olanzapine, ziprasidone	8	56	Allowed	–	–	–	–
Velligan et al. (2002)	Quetiapine, haloperidol	24	34	Allowed	–	SAS	No	Attention, inhibition, verbal memory, psychomotor speed and concentration, cognitive flexibility
Wagner et al. (2005)	Amisulpride, olanzapine	8	36	Allowed	–	SAS	Yes	Declarative memory and composite index (*attention, executive functioning, working memory, verbal learning and memory*)

*EPS, extrapyramidal symptoms; AntiCh, anticholinergic medication; ESRS, extrapyramidal symptom rating scale; SAS, Simpson-Angus scale; AIMS, abnormal involuntary movement scale; SHRS, St. Hans rating scale*.

## Author Contributions

SP and AT wrote the commentary.

## Conflict of Interest Statement

The authors declare that the research was conducted in the absence of any commercial or financial relationships that could be construed as a potential conflict of interest.

## Supplementary Material

The Supplementary Material for this article can be found online at http://journal.frontiersin.org/article/10.3389/fnbeh.2015.00210
