# Supplementary material for: Response to: A Commentary on “Antipsychotic-Induced Parkinsonism is Associated with Working Memory Deficits in Schizophrenia-Spectrum Disorders”
Source: Front Behav Neurosci. 2015 Aug 12;9:210. doi: 10.3389/fnbeh.2015.00210 (PMC4532927; doi:10.3389/fnbeh.2015.00210)
Supplement: Supplementary file 1 [file table_1.docx]

**Supplementary Table 1.** Antipsychotic dosage comparison systems

| **Antipsychotic** | **Chlorpromazine Equivalents** | | | **Daily Defined Dose** ^d^ | **Olanzapine Equivalents** | |
| --- | --- | --- | --- | --- | --- | --- |
|  | Mean Effective Dosage ^a^ | Minimum Effective Dosage ^b^ | Linear equations from expert consensus ^c^ |  | Mean Effective Dosage ^e^ | Minimum Effective Dosage ^b^ |
| Quetiapine | 75 mg/d | 60 mg/d | 175.5 mg/d | 400 mg | 32.3 mg/d | 20 mg/d |
|  |  |  |  |  |  |  |
| Clozapine | 50 mg/d | 120 mg/d | 138.8 mg/d | 300 mg | 30.6 mg/d | 40 mg/d |
|  |  |  |  |  |  |  |
| mg/d = milligram/day; ^a^ ([Woods, 2003](#_ENREF_18)) note that due to the lack of studies investigating clozapine, an earlier CPZ was reported;  ^b^ ([Leucht et al., 2014](#_ENREF_9)); ^c^ ([Andreasen et al., 2010](#_ENREF_1)); ^d^ ([Sweileh et al., 2014](#_ENREF_17)); ^e^ ([Leucht et al., 2015](#_ENREF_8)) | | | | | | |
